# Cloud Model-Based Adaptive Time-Series Information Granulation Algorithm and Its Similarity Measurement

**DOI:** 10.3390/e27020180

**Published:** 2025-02-08

**Authors:** Hailan Chen, Xuedong Gao, Qi Wu, Ruojin Huang

**Affiliations:** 1 School of Business, Sichuan Normal University, Chengdu 610101, China; chenhailan@sicnu.edu.cn (H.C.);; 2 School of Economics and Management, University of Science and Technology Beijing, Beijing 100083, China; 3 School of Finance, Hebei University of Economics and Business, Shijiazhuang 050061, China; kikimama8@hueb.edu.cn

**Keywords:** cloud model, time series, information granulation, similarity measurement, clustering

## Abstract

To efficiently reduce the dimensionality of time series and enhance the efficiency of subsequent data-mining tasks, this study introduces cloud model theory to propose a novel information granulation method and its corresponding similarity measurement. First, we present an information granulation validity index of time series (IGV) based on the entropy and expectation of the cloud model. Taking IGV as the granulation target for time series, an adaptive information granulation algorithm for time series (CMAIG) is proposed, which can transform a time series into a granular time series consisting of several normal clouds without pre-specifying the number of information granules, achieving efficient dimensionality reduction. Then, a new similarity measurement method (CMAIG_ECM) is designed to calculate the similarity between two granular time series. Finally, the hierarchical clustering algorithm based on the proposed time series information granulation method and granular time series similarity measurement method (CMAIG_ECM_HC) is carried out on some UCR datasets and a real stock dataset, and experimental studies demonstrate that CMAIG_ECM_HC has superior performance in clustering time series with different shapes and trends.

## 1. Introduction

Time series, a series of observations recorded in chronological order, is an important type of data widely existing in many fields, including economics, finance, transportation, meteorology, engineering, biology, medicine, etc., [1]. Time-series data mining (TSDM) [2] can extract meaningful knowledge and patterns from time-series data, and has emerged as a significantly important research branch within the data-mining domain. The tasks of TSDM mainly include clustering [3,4,5], classification [6,7], association rule discovery [8], anomaly detection [9], and forecasting [10,11,12], among others. Representation, an essential component of TSDM, plays a crucial role in reducing the high dimensionality of time-series data. Additionally, it can generate a corresponding distance measure, thereby enabling the effective and efficient processing of time-series data [13].

The dimensionality reduction representation methods for time series are primarily divided into two categories: shape-oriented methods and non-shape-oriented methods [14]. Non-shape-oriented methods mainly include statistical approaches such as an autoregressive model (AR), autoregressive moving average model (ARMA), and autoregressive integrated moving average (ARIMA) models, among others [15]. The core of these methodologies lies in converting time-series data into parameters of a fitted model, which are subsequently utilized for clustering or forecasting. However, their restrictive assumptions and parametric nature limit their performance.

Shape-oriented methods aim to extract and represent the shape and trend characteristics of time series. Some common methods include piecewise linear approximation (PLA) [16], piecewise aggregate approximation (PAA) [17], and adaptive piecewise constant approximation (APCA) [18]. These methods typically use mean values or linear functions to represent the characteristics of each segment, failing to reflect more detailed variation information. Furthermore, symbolic aggregate approximation (SAX) [19] is a symbolic representation method based on PAA and, thus, inherits the same limitations as PAA.

Granular computing (GrC) [20] is a novel approach that emulates human problem-solving thought processes and addresses big data analytical tasks. In recent years, it has been applied to time-series analysis. Through a process known as information granulation (IG) [21], the original time series is transformed into a granular time series (GTS) consisting of information granules. The fundamental tasks of time-series information granulation involve the division and representation of information granules. Division methods for information granules typically employ fixed time intervals, while more scholars concentrate on the representation methods of information granules. Common representations of information granules include intervals [22], fuzzy sets [23,24,25,26], and rough sets [27], among others.

Cloud model theory, introduced by Li Deyi in 1995 [28], provides a framework for investigating the uncertain transformation between quantitative data and qualitative concepts, utilizing principles from probability theory and fuzzy mathematics. Cloud model theory has been applied to various domains, including economic evaluation [29], safety and risk assessment [30], decision analysis [31], and data mining [32,33]. As a representation method for information granules, cloud model theory is used to achieve efficient dimensionality reduction in time series. Li et al. [34] presented a time-series segment aggregation approximation method based on the cloud model. Deng et al. [35] provided a two-dimensional normal cloud representation (2D-NCR) approach, which is based on the cloud model theory and transforms raw time series into a sequence of two-dimensional normal cloud models. Si et al. [36] further developed a three-dimensional piecewise cloud representation (TDPCR) method for time-series data. However, these methods require pre-setting the dimensionality of time series, which is challenging to ascertain prior to the reduction process.

In sum, although most of the aforementioned time series representation methods and information granulation methods can effectively extract the trend features of time series, they still have the following shortcomings: (1) The typical partitioning strategy segments a time series into subsequences of equal length, ignoring the variation characteristics of the time series along the time axis, which does not align with the fundamental concept of information granules. (2) Some methods require the input of too many parameters, such as the dimensionality reduction dimension, which is often difficult to determine.

To solve these challenges, this article proposes an adaptive information granulation method for time series based on the cloud model, as well as generating a similarity measurement method for granular time series. The contributions of our work are highlighted as follows:

- An information granulation validity index for time series (IGV) is proposed. This indicator, drawing on internal validity evaluation indicators from clustering, considers two aspects: compactness based on the entropy of cloud model (ComEnCM) and separability based on the expectation of cloud model (SepExCM);
- An adaptive cloud model-based information granulation algorithm (CMAIG) for time series is proposed. We incorporate the IGV index into the algorithm to adaptively determine the number of information granules. Consequently, this algorithm can adaptively segment a time series into several granules represented as normal clouds, constituting a cloud model sequence;
- A novel similarity measurement between two granular time series (CMAIG_ECM) is defined. This method first establishes the matching principles for the cloud model information granules, then applies a cloud model similarity measurement algorithm based on the expected curve to calculate the similarity between the cloud model information granules, and ultimately computes the similarity between two cloud model sequences.

The rest of this article is organized as follows. In Section 2, some related works are reviewed. Section 3 presents an adaptive information granulation algorithm based on the normal cloud model, which does not require a pre-specified number of information granules and can automatically granulate a time series into a series of cloud model information granules. A novel similarity measurement between two cloud model sequences is introduced in Section 4. In Section 5, experiments are represented to illustrate the effectiveness of the time series information granulation method and the similarity measurement method proposed above by performing cluster analysis on time series datasets. Section 6 concludes the study.

## 2. Preliminaries

In this section, we briefly review the fundamental theories of cloud model theory, including normal cloud models and normal cloud generators, which are the basis of this study.

### 2.1. Normal Cloud Model

Cloud model theory can reflect the uncertainty of concepts and reveal the relationship between the fuzziness and randomness of objects. The cloud model is characterized by three parameters: expectation (Ex), entropy (En), and hyper entropy (He). It can be used to represent time series, reflecting the distribution characteristics of the data. Since many phenomena in the real world follow or approximate a normal distribution, the normal cloud [37] is the most common and important model within cloud model theory, which possesses good mathematical properties. Below, we present the relevant concepts and theoretical knowledge of the cloud model and normal cloud [38].

Given *U* is the universe of quantitative data, *C* represents the qualitative concept of *U*. x∈U and *x* is a normal random realization of concept *C*, and the certainty of *x* belonging to *C* is $\mu_{C}(x)\in [0,1]$, which is a stochastic number with stable tendency. The distribution of *x* on *U* is called a cloud, and each *x* is referred to as a cloud droplet.

Three parameters—*Ex*, *En*, and *He*—are utilized to describe clouds. *Ex* represents the mathematical expectation of cloud droplets in *U*. *En* measures the uncertainty of qualitative concept and can be used to describe the cloud span, reflecting the dispersion degree of cloud droplets in *U*. *He* is a measure of the uncertainty of *En* and can be used to describe the thickness of the cloud.

Let *U* be a quantitative universe of discourse represented by precise numerical values, and *C* be a qualitative concept defined by *U*. If the quantitative value *x* ∈ *U*, and *x* is a single random realization of the qualitative concept *C*, generate a random number *En*’, that follows a normal distribution with the expected value of *En* and a standard deviation of *He*. If the random variable *x* satisfies $x∼N(Ex,{En}^{\prime 2})$, where $En\prime ∼N(En,He^{2})$, the degree of certainty of *x* with respect to *C* satisfies

(1) $$\mu_{C}(x)=e^{-\frac{{\left(x-Ex\right)}^{2}}{2{\left(En^{\prime }\right)}^{2}}}$$

The distribution of *x* on *U* is called normal cloud.

**Figure 1:**
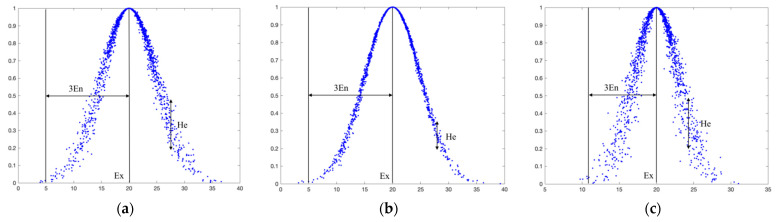
Normal cloud models with different parameters. (**a**) (*Ex*, *En*, *He*) = (20, 5, 0.5); (**b**) (*Ex*, *En*, *He*) = (20, 5, 0.2); (**c**) (*Ex*, *En*, *He*) = (20, 3, 0.5).

Figure 1 displays examples of three normal cloud models with different parameters, allowing us to understand the roles of three parameters (*Ex*, *En*, *He*) of the cloud model, and how they are represented visually. Moreover, similar to the 3*σ* rule of the normal distribution, the normal cloud also follows a 3*En* rule. Most of the cloud droplets fall within the interval [*Ex* − 3*En*, *Ex* + 3*En*], with a small portion of the cloud droplets located outside this interval. Thus, ignoring them does not affect the overall shape characteristics of the normal cloud.

The cloud model has no definite edges but has an overall shape. Li et al. [37] proposed using the expectation curve of the cloud to describe the contour characteristics of the cloud model. The expectation curve of the normal cloud is defined as follows:

(2) $$y=e^{-\frac{{\left(x-Ex\right)}^{2}}{2En^{2}}}$$

**Figure 2:**
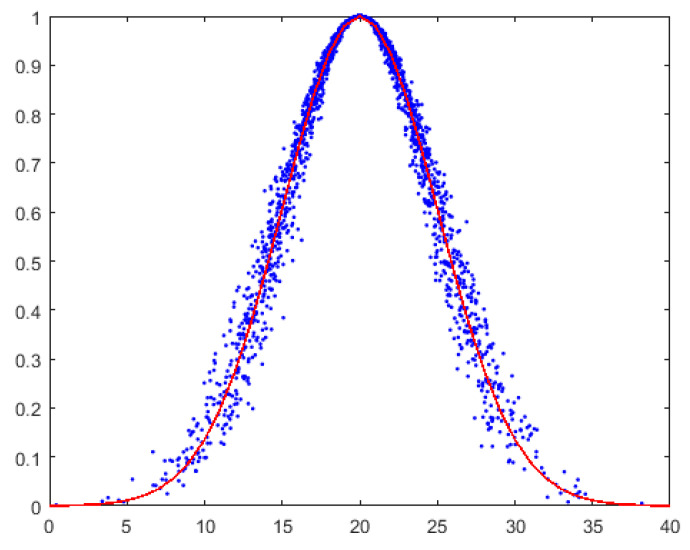
Expectation curve of a normal cloud model.

The expectation curve passes smoothly through the “middle” of the cloud droplets. It outlines the overall “contour” of the cloud and is the “skeleton” of the set of cloud droplets. All cloud droplets fluctuate randomly near the expectation curve, and the magnitude of the fluctuation is controlled by *He*. As shown in Figure 2, the blue dots represent cloud droplets, and the red line is the expectation curve of the normal cloud model. It can be observed from the figure that all cloud droplets are randomly distributed around the expectation curve of the normal cloud. Consequently, the expectation curve of the normal cloud can accurately reflect the important geometric characteristics of the normal cloud.

### 2.2. Normal Cloud Generator

The Cloud Generator (CG) [28] refers to the algorithm for generating cloud models. Through the cloud generator, the mutual transformation between qualitative concepts and quantitative values can be established, which mainly includes the forward normal cloud generator (FNCG) [39] and the backward normal cloud generator (BNCG) [40].

Algorithm 1 is the FNCG algorithm, which can generate a collection of precise data (cloud droplets) with a certain degree of randomness based on the three parameters (*Ex*, *En*, *He*) of a qualitative concept. Algorithm 2, the BNCG algorithm, transforms quantitative data samples (cloud droplets) into qualitative concepts represented by *Ex*, *En,* and *He*.
**Algorithm 1: Forward Normal Cloud Generator (FNCG)****Input:** Cloud model parameters *Ex*, *En*, *He* and the number of cloud droplets *n* **Output:** Cloud droplets collection [x(i),y(i)](i=1,2,⋯,n)1. $En^{\prime }=randn(1)He+En$, where *randn*(1) represents the generation of a random number following a normal distribution with a mean of 0 and a variance of 1;2. **for**
*i* = 1 to *n* **do** 3.       x(i)=randn(1)En′+Ex;4. $\;\;\;\;\;\;y(i)=e^{-\frac{{\left(x(i)-Ex\right)}^{2}}{2En^{\prime 2}}}$;5. end **for** 6. return cloud droplets collection [x(i),y(i)].


**Algorithm 2: Backward Normal Cloud Generator (BNCG)**
**Input:** Cloud droplets X=x(i)(i=1,2,⋯,n) **Output:** Cloud model parameters *Ex*, *En*, *He*1. Ex=Mean(X), where Mean(•) is the average value function; 2. $En=\sqrt{\frac{\pi }{2}}Mean(\left|X-Ex\right|)$; 3. $He=\sqrt{Var(x)-En^{2}}$, where Var(•) is the variance function; 4. return *Ex*, *En*, *He*

The normal cloud model is universal [37]. Specifically, it does not require the data to strictly follow a normal distribution, but rather a relatively loose pan-normal distribution. Through the BNCG algorithm, a vague and approximate description of the data can be obtained, making it less susceptible to noise within the data. In many fields of natural and social science, the data usually approximately follow a pan-normal distribution and are suitable for processing using the normal cloud generator [38].

## 3. Adaptive Time-Series Information Granulation Algorithm Based on the Cloud Model

In this section, we propose an adaptive information granulation algorithm for time series based on the cloud model, which does not require a pre-specified number of information granules and can automatically granulate an original time series into a series of cloud model granules. Section 3.1 introduces an information granulation validity index of time series (IGV) based on the entropy and expectation of the cloud model, which is used to evaluate the granulation results corresponding to different numbers of information granules. In Section 3.2, we incorporate this index into the granulation algorithm, proposing an adaptive information granulation algorithm based on the cloud model (CMAIG).

### 3.1. Information Granulation Validity Index of Time Series

Information granulation of time series involves dividing the time series into a series of information granules, such that the data characteristics within each information granule are approximately similar to each other, while the data characteristics between different information granules tend to be distinct. This is essentially the same as the goal of clustering, which is to have similarity within a cluster and dissimilar between clusters. Therefore, we propose an effectiveness evaluation index for cloud model-based information granulation, drawing on the internal validity evaluation index of clustering. This index also consists of two parts: an intra-granule compactness measurement and inter-granule separability measurement.

**Definition** **1.** *Compactness based on entropy of cloud model (ComEnCM).*

For a time series *Q* in the dataset, after the information granulation operation, it is divided into *w* subsequences $Q=\left\{IG_{1},IG_{2},\ldots ,IG_{w}\right\}$. Each subsequence is considered an information granule and is represented by the cloud model $IG_{i}=(Ex_{i},En_{i},He_{i})$. The intra-granule compactness of the granulated time series is expressed by the following formula:

(3) $$ComEnCM(w)=\frac{1}{w}\sum_{i=1}^{w}En_{i}$$

where *w* represents the number of information granules after granulation, and *En_i_* denotes the entropy of the *i*-th cloud model information granule.

Since *En* of the cloud model reflects the dispersion of cloud droplets, it can be used to evaluate the similarity of data within the information granule. That is, the smaller *En*, the more stable the data within the information granule, and the more similar they are to each other. For a cloud model sequence, the smaller *ComEnCM*, the better the granulation effect.

**Definition** **2.** *Separability based on expectation of cloud model (SepExCM).*

The inter-granule separability of the time series after granulation is expressed by the following formula:

(4) $$SepExCM(w)=\frac{1}{w-1}\sum_{i=1}^{w-1}\left|Ex_{i+1}\right.-\left.Ex_{i}\right|$$

where *w* represents the number of information granules after granulation, and *Ex_i_* denotes the expectation of the *i*-th cloud model information granule.

Since the data of a time series have a sequential order on the time axis, the granulated information granules also have a sequential order. Unlike the calculation method for inter-cluster separability, it is meaningless to calculate the distances between each information granule. Therefore, we only calculate the distances between adjacent information granules. Here, the expectation of the cloud model is used to represent a certain information granule, and the distance between two adjacent information granules is represented by the difference in their expectations. Obviously, the greater *SepExCM*, the better the granulation effect.

**Definition** **3.** *Information granulation validity index of time series (IGV).*

Based on *ComEnCM* and *SepExCM*, we construct an information granulation validity index of time series. The calculation formula is as follows:

(5) $$IGV(w)=\frac{SepExCM(w)}{ComEnCM(w)}=\frac{\frac{1}{w-1}\sum_{i=1}^{w-1}\left|Ex_{i+1}\right.-\left.Ex_{i}\right|}{\frac{1}{w}\sum_{i=1}^{w}En_{i}}=\frac{w\sum_{i=1}^{w-1}\left|Ex_{i+1}\right.-\left.Ex_{i}\right|}{(w-1)\sum_{i=1}^{w}En_{i}}$$

where *w* represents the number of information granules after granulation, and *En_i_* and *Ex_i_*, respectively, denote the entropy and expectation of the *i*-th cloud model information granule.

The smaller *ComEnCM*, the less variation there is within an information granule, meaning they are more similar. Conversely, the larger *SepExCM*, the more distinct the information granules are from each other, indicating less similarity. Hence, a higher *IGV* signifies a more effective information granulation process.

### 3.2. Adaptive Time-Series Information Granulation Algorithm

Taking the proposed index *IGV* as the granulation target for the time series, we propose an adaptive information granulation algorithm for time series that automatically determines the number of information granules *w*.

**Figure 3:**
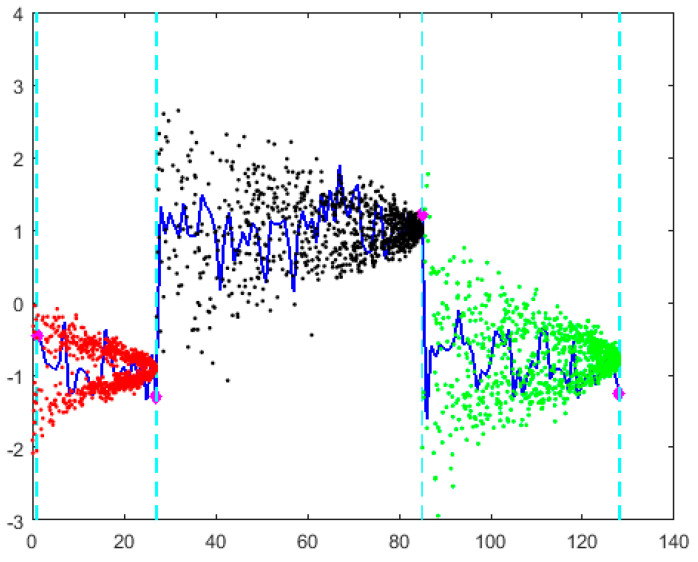
Time series partition using Algorithm 3.

Take a time series from CBF dataset as an example, and apply Algorithm 3 to perform information granulation, as shown in Figure 3. This time series is divided into three subsequences, which aligns with the actual feature partition of the original time series. Then, each subsequence is referred to as an information granule, represented by cloud model parameters, as shown in Table 1.
**Algorithm 3: Adaptive Time-Series Information Granulation Algorithm Based on Cloud Model (CMAIG)****Input:** Time Series $X=\left\{x_{1},x_{2},\ldots ,x_{n}\right\}$ **Output:** Cloud model sequence $C_{x}=\left\{c_{x1},c_{x2},\ldots ,c_{xw}\right\}$, where $c_{x_{i}}=\left[t_{si},t_{ei},Ex_{i},En_{i},He_{i}\right]$1. Initialize time series *X*. Use a matrix $V_{w\times 5}$ to record the cloud model information of each segment, where V(k,1:2) records the start timestamp *t_s_* and the end timestamp *t_e_* of the *k*-th cloud model information granule, and V(k,3:5) records the three parameters (*Ex*, *En*, *He*) of the *k*-th cloud model information granule. Here, *Ex*, *En* and *He* are obtained through Algorithm 2, *k* is the current number of information granules, and set the initial *k* = 1, that is, V(k,1:2)=[1:n], V(k,3:5)=back_cloud(X); 2. For V(:,4), which is the fourth column of *V*, locate its maximum value of V(:,4)(V(:,2)−V(:,1)), and denote the subsequence corresponding to this maximum value as *k*_0_; 3. Let $t_{1}=V(k_{0},1)$, $t_{2}=V(k_{0},2)$, and search for *t*_0_ ($t_{1}<t_{0}<t_{2}$) in the sebsequence $X(t_{1}:t_{2})$. Calculate $L(1:2)=[t_{1}:t_{0}]$, $L(3:5)=back\_cloud(X(t_{1}:t_{0}))$ and $R(1:2)=[t_{0}:t_{2}]$, $R(3:5)=back\_cloud(X(t_{0}:t_{2}))$. Maximize the following expression $\Delta En=V(k_{0},4)(V(k_{0},2)-V(k_{0},1))-(L(4)(L(2)-L(1))+R(4)(R(2)-R(1)))$; 4. Calculate the current granulation index IGV(k) according to Equation (5). If IGV(k)<IGV(k−1), then stop and output *C_x_* of w=k−1; otherwise, let $V(k_{0},:)=L$, $V(k_{0}+1,:)=R$, k=k+1, return to step 2.

In summary, the proposed algorithm CMAIG can identify and represent the shape or trend of time series, and the parameters of the cloud model can effectively describe the data distribution within each information granule. By applying CMAIG, the original time series is transformed into a granular time series, also known as a cloud model sequence.

## 4. Similarity Measurement Method for Granular Time Series

In this section, we propose a novel similarity measurement method between two granular time series. Section 4.1 introduces the matching method for two granular time series consisting of cloud model information granules (CMIGs). In Section 4.2, the cloud model similarity measurement based on the expected curve is used to calculate the similarity between a matched pair of cloud models. Section 4.3 presents the method for calculating the similarity of two granular time series.

### 4.1. Information Granules Matching Method of Granular Time Series

**Figure 4:**
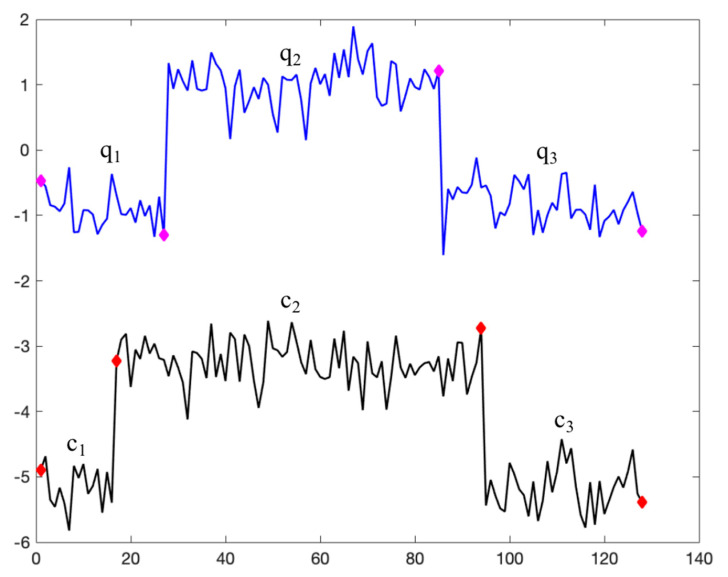
Example of two granular time series after operating CMAIG.

After performing CMAIG on different time series, the resulting granular time series typically exhibit the following phenomena: (1) varying numbers of information granules; and (2) varying sizes of information granule time windows. As shown in Figure 4, taking two time series from the CBF dataset as an example, one of the time series has been translated along the vertical axis for ease of observation. Both time series have been divided into three segments using Algorithm 3. Although the number of information granules is the same, the corresponding time points and lengths of the time windows for information granules differ. The specific CMIGs representation of two time series is shown in Table 2.

We define the matching principle for CMIGs as follows: all information granules that intersect with the time window of a given information granule are considered matches. Therefore, the pairs of matched CMIGs between granular time series *Q^c^* and *C^c^* are {*q*_1_, *c*_1_}, {*q*_1_, *c*_2_}, {*q*_2_, *c*_2_}, {*q*_3_, *c*_2_}, {*q*_3_, *c*_3_}, with the specific information shown in Table 3.

According to the matching principle, two granular time series are transformed into several matching pairs of CMIGs.

### 4.2. Similarity Measurement of CMIGs Based on Cloud Model Expected Curve

In this section, we focus on the similarity measurement between each matching pair of CMIGs. Since the expected curve of a normal cloud can accurately reflect the important geometric characteristics of the normal cloud, it is usually introduced to calculate the similarity between each pair of CMIGs [41,42].

**Figure 5:**
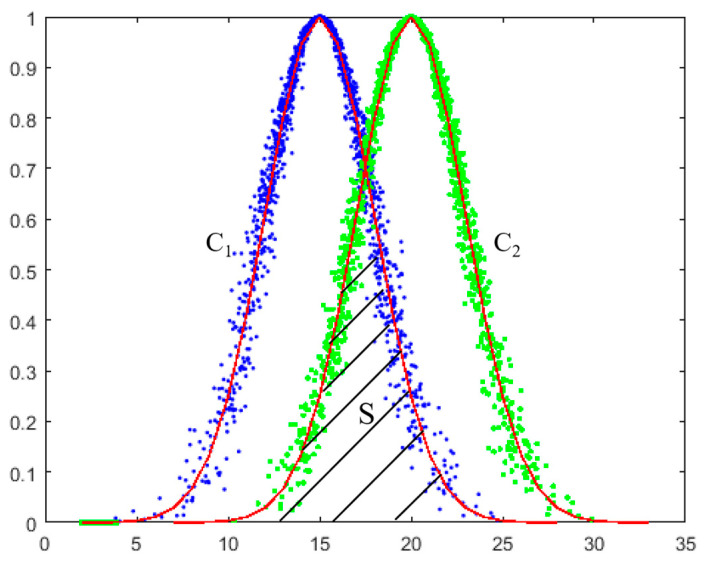
The intersection of cloud model $C_{1}(15,3,0.35)\;\mathrm{and}C_{2}(20,3,0.3)$.

As shown in Figure 5, we plot the expected curves of two cloud models, $C_{1}(15,3,0.35)$ and $C_{2}(20,3,0.3)$. The similarity between two cloud models can be represented by the area of the overlapping intersection of their expected curves [41], which is depicted as the shaded area in the figures. Next, we calculate the area of the overlapping intersection between the expectation curves of two cloud models.

Based on the three parameters of the cloud model, using Equation (2), we can derive the expression for its expected curve. The area of the intersection region is represented by solving the integral of two expected curves. Assuming the abscissa of the intersection point of two expected curves is *x*_0_, then the area of the intersection is given by

(6) $$S=\int_{-\infty }^{x_{0}}y_{2}(x)dx+\int_{x_{0}}^{+\infty }y_{1}(x)dx$$

where $y_{1}(x)$ and $y_{2}(x)$ represent the expected curve expressions of the normal cloud model *C*_1_ and *C*_2_, respectively.

Obviously, Equation (6) cannot be computed analytically and is reduced to integrals with respect to the standard normal law. From Equation (2), it is evident that the expression of the expected curve is similar to the probability density function of the normal distribution, and thus it can be transformed as follows:

(7) $$y=\sqrt{2\pi }En\frac{1}{\sqrt{2\pi }En}e^{-\frac{{\left(x-Ex\right)}^{2}}{2En^{2}}}=\sqrt{2\pi }Enf(x)$$

where *f*(*x*) represents the probability density function of the normal distribution. Using the properties of the normal distribution, Equation (6) can be transformed as follows:

(8) $$S=\int_{-\infty }^{x_{0}}y_{2}(x)dx+\int_{x_{0}}^{+\infty }y_{1}(x)dx=\sqrt{2\pi }En_{2}\int_{-\infty }^{x_{0}}y_{2}(x)dx+\sqrt{2\pi }En_{1}\int_{x_{0}}^{+\infty }y_{1}(x)dx$$

Furthermore, converting the general normal distribution in Equation (8) to the standard normal distribution results in the following:

(9) $$\begin{array}{l} S=\sqrt{2\pi }En_{2}\int_{-\infty }^{x_{0}}y_{2}(x)dx+\sqrt{2\pi }En_{1}\int_{x_{0}}^{+\infty }y_{1}(x)dx\\ \;\;=\sqrt{2\pi }En_{2}\int_{-\infty }^{z_{2}}\phi (x)dx+\sqrt{2\pi }En_{1}(1-\int_{-\infty }^{z_{1}}\phi (x)dx) \end{array}$$

where $z_{1}=\frac{x_{0}-Ex_{1}}{En_{1}}$, $z_{2}=\frac{x_{0}-Ex_{2}}{En_{2}}$, ϕ(x) represents the probability density function of the standard normal distribution. When the value of *x*_0_ is known, the values of *z*_1_ and *z*_2_ can be determined. Then, by consulting the standard normal distribution table and substituting the value of ϕ(x) into Equation (9), the intersection area *S* of two expected curves can be obtained.

We consider the expected curves of our two cloud models as $y_{1}(x)=e^{-\frac{{\left(x-Ex_{1}\right)}^{2}}{{{2En}_{1}}^{2}}}$ and $y_{2}(x)=e^{-\frac{{\left(x-Ex_{2}\right)}^{2}}{{{2En}_{2}}^{2}}}$. When the two curves intersect, $y_{1}(x)=y_{2}(x)$ holds at the intersection point, that is, $\left|z_{1}\right|=\left|z_{2}\right|$. By substituting the expressions of *z*_1_ and *z*_2_, we can derive the expressions for the intersection points $x_{0}^{\left(1\right)}=\frac{Ex_{2}En_{1}-Ex_{1}En_{2}}{En_{1}-En_{2}}$ and $x_{0}^{\left(2\right)}=\frac{Ex_{1}En_{2}+Ex_{2}En_{1}}{En_{1}+En_{2}}$.

Similarly to the 3*σ* rule of the normal distribution, the normal cloud also follows a 3*En* rule, which means that 99.74% of the cloud droplets fall within the interval [Ex−3En,Ex+3En]. Therefore, when calculating the similarity of the normal cloud model, it is possible to consider only the distribution of cloud droplets within this interval.

Given two cloud models $C_{1}(Ex_{1},En_{1},He_{1})$ and $C_{2}(Ex_{2},En_{2},He_{2})$, assuming $Ex_{1}\leq Ex_{2}$, based on the distribution of the intersection points $x_{0}^{\left(1\right)}$ and $x_{0}^{\left(2\right)}$, there are three scenarios for the intersection of the expected curves of the two cloud models. Consequently, the solution for the area *S* of the intersection part exists as follows.

(1) Scenario One: no intersection points in [Ex−3En,Ex+3En]

**Figure 6:**
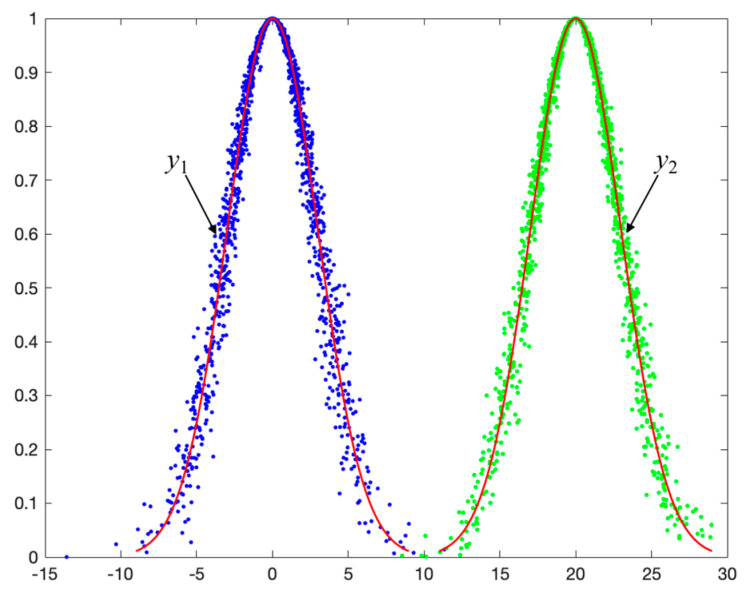
Example of scenario one: the intersection of cloud model $C_{1}(0,3,0.35)\;\mathrm{and}C_{2}(20,3,0.3)$.

As shown in Figure 6, when $x_{0}^{\left(1\right)}$ and $x_{0}^{\left(2\right)}$ both fall outside the interval [Ex−3En,Ex+3En], the cloud droplets between the two intersection points can be ignored, and the intersection area is denoted as *S* = 0.

(2) Scenario Two: one intersection point in [Ex−3En,Ex+3En]

**Figure 7:**
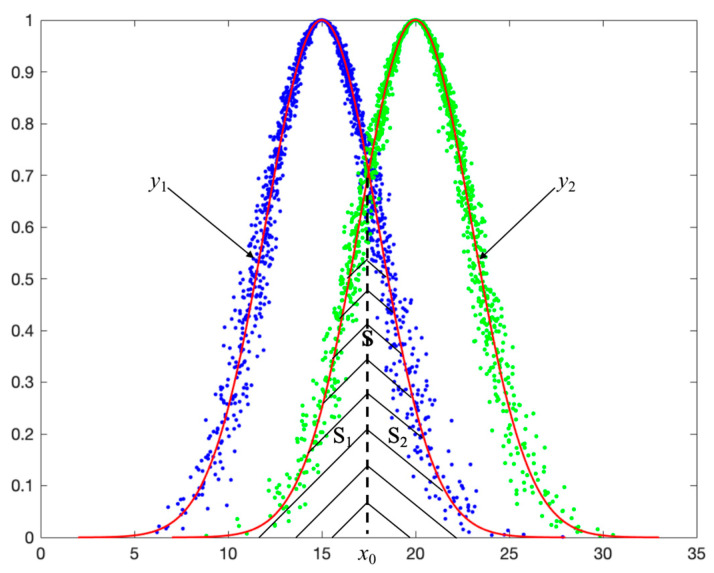
Example of scenario two: the intersection of cloud model $C_{1}(15,3,0.35)\;\mathrm{and}C_{2}(20,3,0.3)$.

As shown in Figure 7, when one of the points $x_{0}^{\left(1\right)}$ and $x_{0}^{\left(2\right)}$ falls within the interval [Ex−3En,Ex+3En], the intersection area consists of two parts, denoted as *S* = *S*_1_ + *S*_2_.

(3) Scenario Three: two intersection points in [Ex−3En,Ex+3En]

**Figure 8:**
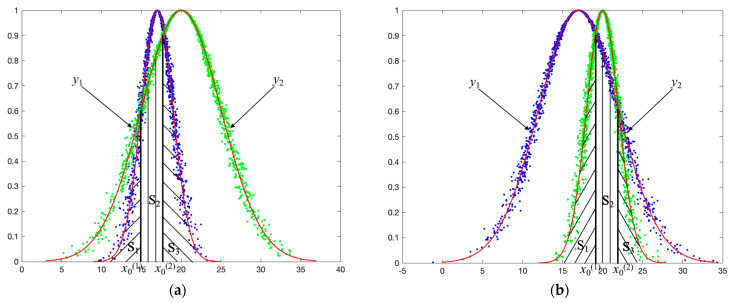
Example of scenario three. (**a**) $En_{1}\leq En_{2}$: the intersection of cloud model $C_{1}(17,2,0.25)$ and $C_{2}(20,5,0.3)$; (**b**) $En_{1}>En_{2}$: the intersection of cloud model $C_{1}(17,5,0.3)$ and $C_{2}(20,2,0.25)$.

When $x_{0}^{\left(1\right)}$ and $x_{0}^{\left(2\right)}$ both fall within the interval [Ex−3En,Ex+3En], the intersection area is composed of three parts, that is *S* = *S*_1_ + *S*_2_ + *S*_3_. Since the entropy of the two cloud models differs, there are two scenarios as shown in Figure 8. Below is a detailed analysis of these two scenarios.

As shown in (a) of Figure 8, if $En_{1}\leq En_{2}$, then in the three parts of *S*, *S*_1_ and *S*_3_ can be obtained from the expected curve *y*_1_ of cloud model *C*_1_, and *S*_2_ can be obtained from the expected curve *y*_2_ of cloud model *C*_2_.

As shown in (b) of Figure 8, if $En_{1}>En_{2}$, then in the three parts of *S*, *S*_1_ and *S*_3_ can be obtained from the expected curve *y*_2_ of cloud model *C*_2_, and *S*_2_ can be obtained from the expected curve *y*_1_ of cloud model *C*_1_.

Assuming $En_{1}\leq En_{2}$ and $x_{0}^{\left(1\right)}\leq x_{0}^{\left(2\right)}$, then the solutions of *S*_1_, *S*_2_ and *S*_3_ are as follows.

*S*_1_ can be calculated through the expected curve *y*_1_ of cloud model *C*_1_:

(10) $$S_{1}=\sqrt{2\pi }En_{1}\int_{-\infty }^{x_{0}^{\left(1\right)}}f_{1}(x)dx=\sqrt{2\pi }En_{1}\int_{-\infty }^{z_{1}^{\left(1\right)}}\phi (x)dx$$

where $z_{1}^{\left(1\right)}=\frac{x_{0}^{\left(1\right)}-Ex_{1}}{En_{1}}$.

*S*_2_ can be calculated through the expected curve *y*_2_ of cloud model *C*_2_:

(11) $$\begin{array}{l} S_{2}=\sqrt{2\pi }En_{2}\int_{x_{0}^{\left(1\right)}}^{x_{0}^{\left(2\right)}}f_{2}(x)dx=\sqrt{2\pi }En_{2}(\int_{-\infty }^{x_{0}^{\left(2\right)}}f_{2}(x)dx-\int_{-\infty }^{x_{0}^{\left(1\right)}}f_{2}(x)dx)\\ \;\;\;\;=\sqrt{2\pi }En_{2}(\int_{-\infty }^{z_{2}^{\left(2\right)}}\phi (x)dx-\int_{-\infty }^{z_{2}^{\left(1\right)}}\phi (x)dx) \end{array}$$

where $z_{2}^{\left(2\right)}=\frac{x_{0}^{\left(2\right)}-Ex_{2}}{En_{2}}$, $z_{2}^{\left(1\right)}=\frac{x_{0}^{\left(1\right)}-Ex_{2}}{En_{2}}$.

*S*_3_ can be calculated through the expected curve *y*_1_ of cloud model *C*_1_:

(12) $$\begin{array}{l} S_{3}=\sqrt{2\pi }En_{1}\int_{x_{0}^{\left(2\right)}}^{+\infty }f_{1}(x)dx=\sqrt{2\pi }En_{1}(1-\int_{-\infty }^{x_{0}^{\left(2\right)}}f_{1}(x)dx)\\ \;\;\;\;=\sqrt{2\pi }En_{1}(1-\int_{-\infty }^{z_{1}^{\left(2\right)}}\phi (x)dx) \end{array}$$

where $z_{1}^{\left(2\right)}=\frac{x_{0}^{\left(2\right)}-Ex_{1}}{En_{1}}$.

By consulting the standard normal distribution table and using the aforementioned formulas to calculate the values of *S*_1_, *S*_2_, and *S*_3_ separately, we obtain *S* = *S*_1_ + *S*_2_ + *S*_3_. To facilitate the comparison of similarity between different cloud models, we standardize *S* to obtain the similarity measurement based on the cloud model expected curve (ECM) as follows:

(13) $$sim_{ECM}(C_{1},C_{2})=\frac{2S}{\sqrt{2\pi }(En_{1}+En_{2})}$$

where $\sqrt{2\pi }En_{1}$ represents the area between the expected curve *y*_1_ of cloud model *C*_1_ and the horizontal axis, and $\sqrt{2\pi }En_{2}$ denotes the area between the expected curve *y*_2_ of the cloud model *C*_2_ and the horizontal axis. Clearly, $sim_{ECM}(C_{1},C_{2})\in [0,1]$, the larger the values of $sim_{ECM}(C_{1},C_{2})$, the greater the similarity between the two cloud models.

If $En_{1}>En_{2}$, the same method can be applied to find the solution. In summary, we outline the steps of the similarity measurement algorithm of CMIGs based on cloud model expected curve as follows.

For each matching pair of CMIGs, Algorithm 4 can be applied to obtain their similarity.
**Algorithm 4: Similarity Measurement Algorithm of CMIGs Based on Cloud Model Expected Curve (ECM**)**Input:** Two normal cloud models $C_{1}=\left[Ex_{1},En_{1},He_{1}\right]$ and $C_{2}=\left[Ex_{2},En_{2},He_{2}\right]$ **Output:** The similarity $sim_{ECM}(C_{1},C_{2})$ of *C*_1_ and *C*_2_1. Assume $En_{1}\leq En_{2}$, according to Equation (2), we solve for the expected curves of two normal cloud models *C*_1_ and *C*_2_, and calculate $x_{0}^{\left(1\right)}$ and $x_{0}^{\left(2\right)}$, let $x_{0}^{\left(1\right)}\leq x_{0}^{\left(2\right)}$; 2. If $x_{0}^{\left(1\right)}\leq \min(Ex_{1}-3En_{1},Ex_{2}-3En_{2})$ and $x_{0}^{\left(2\right)}\geq \max(Ex_{1}+3En_{1},Ex_{2}+3En_{2})$, then *S* = 0 and stop; otherwise, proceed to Step 3; 3. If $x_{0}^{\left(1\right)}\geq \max(Ex_{1}-3En_{1},Ex_{2}-3En_{2})$ and $x_{0}^{\left(2\right)}\leq \min(Ex_{1}+3En_{1},Ex_{2}+3En_{2})$, then assess the sizes of *En*_1_ and *En*_2_, and solve for *S*_1_, *S*_2_, and *S*_3_ using Equations (10), (11), and (12), respectively, thus obtaining *S* = *S*_1_ + *S*_2_ + *S*_3_; otherwise, proceed to Step 4; 4. If one of the points $x_{0}^{\left(1\right)}$ and $x_{0}^{\left(2\right)}$ falls within the interval [Ex−3En,Ex+3En], then *S* = *S*_1_ + *S*_2_; 5. Substitute *S* into Equation (13) to calculate the similarity $sim_{ECM}(C_{1},C_{2})$.

### 4.3. Similarity Measurement for Cloud Model Sequences

In Section 4.1, we proposed a matching method for CMIGs. Section 4.2 provided a method for calculating the similarity between two cloud models, which can be used to calculate the similarity between each matching pair of CMIGs. In this section, based on the aforementioned methods, we present how to calculate the similarity between two cloud model sequences.

Firstly, we provide the formula for calculating the similarity between two cloud model sequences:

(14) $$Sim(Q^{c},C^{c})=\sqrt{\frac{1}{w^{\prime }}\sum_{i=1}^{w^{\prime }}k_{i}sim_{i}}$$

where *Q^c^* and *C^c^* are cloud model sequences consisting of CMIGs after applying CMAIG, *w’* represents the number of matching pairs of CMIGs, and *sim_i_* indicates the similarity between the *i*-th pair of CMIGs, which is calculated using Equation (13). *k_i_* denotes the time window coverage between the *i*-th pair of CMIGs, and the calculation formula is as follows:

(15) $$k_{i}=\frac{card\left(tw_{q}\cap tw_{c}\right)}{\max\left\{card\left(tw_{q}\right),card\left(tw_{c}\right)\right\}}$$

where $tw_{q}=\left\{t\in T_{Q}\left|t_{q}^{s}\leq t\leq t_{q}^{e}\right.\right\}$ represents the time window of cloud model *q*, *T_Q_* indicates the set of timestamps for time series *Q*, and $t_{q}^{s}$ and $t_{q}^{e}$ denote the start and end timestamps of cloud model *q*, respectively. Similarly, $tw_{c}=\left\{t\in T_{C}\left|t_{c}^{s}\leq t\leq t_{c}^{e}\right.\right\}$ represents the time window of cloud model *c*, *T_C_* indicates the set of timestamps for time series *C*, and $t_{c}^{s}$ and $t_{c}^{e}$ denote the start and end timestamps of cloud model *c*, respectively. Define $card\left(•\right)$ to represent the number of elements in a set. Then, $card\left(tw_{q}\cap tw_{c}\right)$ indicates the number of elements in the intersection of the time windows of cloud models *q* and *c*, and $\max\left\{card\left(tw_{q}\right),card\left(tw_{c}\right)\right\}$ denotes the maximum number of elements contained in the time windows of cloud models *q* and *c*.

Then, we propose the similarity measurement algorithm for cloud model sequences.

In summary, Algorithm 3 presented in Section 3 can transform the original time series into granular time series consisting of CMIGs. By applying Algorithm 5, which is proposed in this section, we obtain the similarity between two granular time series, which can represent the similarity between the original time series.
**Algorithm 5: Similarity Measurement Algorithm for Cloud Model Sequences (CMAIG_ECM)****Input:** Two cloud model sequences $Q^{c}=\left\{q_{1}^{c},q_{2}^{c},\ldots ,q_{w_{1}}^{c}\right\}$ and $C^{c}=\left\{c_{1}^{c},c_{2}^{c},\ldots ,c_{w_{2}}^{c}\right\}$ **Output:** The similarity $Sim(Q^{c},C^{c})$ of *Q^c^* and *C^c^*1. Apply the information granules matching method to match *Q^c^* and *C^c^*, then, obtain several matching pairs of CMIGs; 2. For each matching pair of CMIGs, calculate the time window coverage degree *k_i_* according to Equation (15); 3. Calculate the similarity $sim_{ECM}(q_{i},c_{j})$ between each matching pair of CMIGs using Algorithm 4; 4. Substitute *k_i_* and $sim_{ECM}(q_{i},c_{j})$ into Equation (14) to calculate the similarity $Sim(Q^{c},C^{c})$.

## 5. Experiments

In this section, a series of experiments are conducted to illustrate the performance of the proposed methods. In Section 5.1, we design the experimental strategy where the clustering algorithm is employed to validate the effectiveness of the proposed information granulation method and the similarity measurement method. In Section 5.2, some evaluation metrics are introduced to assess the information granulation results and the clustering results. Section 5.3 and Section 5.4 operate on some datasets from the UCR Time Series Classification Archive [43] for experiments, comparing the proposed algorithm with existing related algorithms. Section 5.5 uses the real stock dataset [44] from China’s Shanghai and Shenzhen A-Share Market for practical application.

### 5.1. Experimental Strategy

To verify the effectiveness of the proposed methods, we perform the clustering algorithm on time series datasets and then analyze the clustering results. Common clustering techniques encompass a segmentation clustering method, hierarchical clustering method, density-based clustering method, and grid-based clustering method [45]. Hierarchical clustering, a prototype-based approach, endeavors to create a multi-level dataset partitioning that culminates in a tree-like structure. This technique facilitates the visual interpretation of clustering results through the construction of a dendrogram. Additionally, it eliminates the prerequisite of predetermining the number of clusters prior to the clustering process.

**Figure 9:**
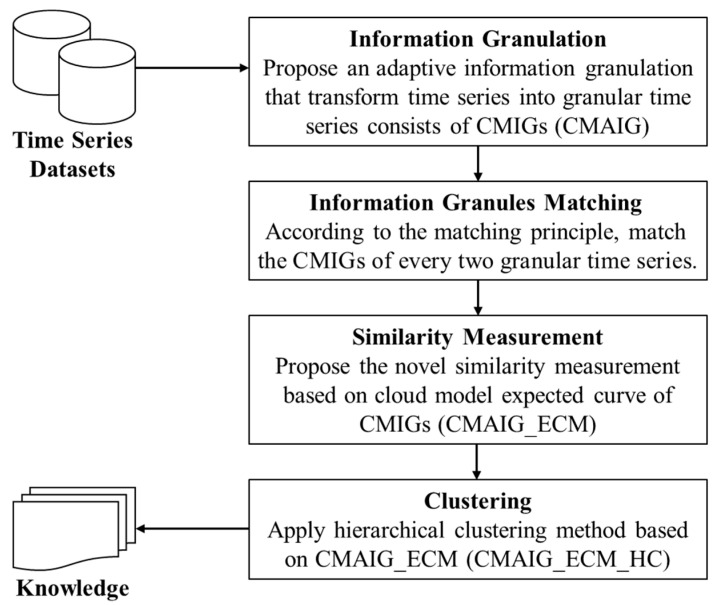
The framework of experimental strategy.

Considering the above advantages, a hierarchical clustering algorithm is selected as the clustering method for the experiments in this paper. We design the experimental strategy as follows: First, the proposed information granulation method (CMAIG) is applied to transform the original time series into a granular time series; then, we calculate the similarity between any two granular time series using the proposed similarity measurement (CMAIG_ECM); finally, the hierarchical clustering algorithm based on the proposed similarity measurement (CMAIG_ECM_HC) is operated on the dataset. We summarize the framework of the designed experimental strategy as illustrated by Figure 9.

### 5.2. Evaluation Metrics

#### 5.2.1. Dimensionality Reduction Evaluation Metric

To measure the degree of dimensionality reduction in the original time series by the information granulation method, the data compression rate (CR) is used as an evaluation metric. Its calculation formula is as follows:

(16) $$CR=\frac{w}{m}\times 100\%$$

where *m* represents the amount of data in the original time series, and *w* indicates the number of information granules after information granulation. Clearly, the smaller the *CR*, the greater the degree of data dimensionality reduction.

#### 5.2.2. Clustering Validity Evaluation Metric

*F*-measure is typically used to evaluate the performance of clustering methods [46]. For any two sample points, when they are in the same class both before and after the clustering process, these are referred to as positive events *T*. On the contrary, if two sample points belong to the same class prior to clustering yet end up in different classes after the clustering process, then they are named negative events *F*. And the value of *F*-measure is calculated by

(17) $$F-\mathrm{Measure}=\frac{2\times Precision\times Recall}{Precision+Recall}$$

where

(18) $$Precision=\frac{TP}{TP+FP}$$

(19) $$Recall=\frac{TP}{TP+FN}$$

*TP* (True Positive) represents the quantity of sample pairs that remain in the same class both prior to and after the clustering operation. *FN* (False Negative) indicates the number of sample pairs that were in the same class before clustering took place but are not placed in the same cluster after the clustering process. *FP* (False Positive) stands for the number of sample pairs that did not belong to the same class before clustering yet are grouped into the same cluster after the clustering process. *TN* (True Negative) refers to the number of sample pairs that are not in the same class either before or after the clustering process. The value of *F*-measure ranges from 0 to 1. The larger the value is, the higher the accuracy of the clustering results will be.

### 5.3. Experiment A on CBF Dataset

**Figure 10:**
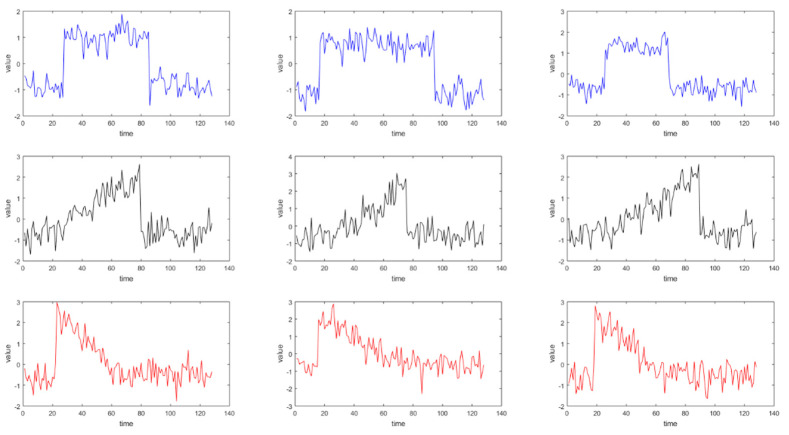
Nine time series from three classes of CBF dataset.

**Figure 11:**
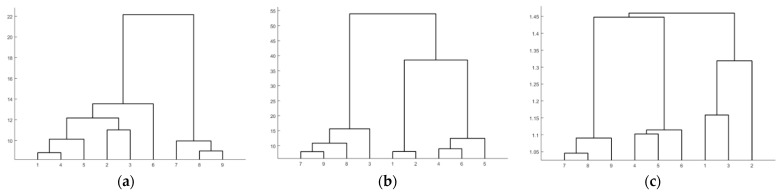
The clustering results of different methods. (**a**) ED_HC; (**b**) DTW_HC; (**c**) CMAIG_ECM_HC.

Experiment A is conducted on the CBF dataset from the UCR Time Series Classification Archive. As depicted in Figure 10, the experiment involves nine time series selected from three different classes. Then, we apply the CMAIG_ECM_HC method to the experimental data. The comparison methods include hierarchical clustering based on Euclidean distance (ED_HC) and hierarchical clustering based on Dynamic Time Warping distance (DTW_HC). In addition, the clustering results of different methods are shown in Figure 11.

As illustrated in Figure 11, the clustering outcomes of ED_HC are {1,2,3,4,5}, {6}, and {7,8,9}. For DTW_HC, the clusters are {1,2}, {4,5,6}, and {3,7,8,9}. In contrast, the clustering result of the CMAIG_ECM_HC method proposed in this article is {1,2,3}, {4,5,6}, and {7,8,9}, which aligns with the actual class divisions. From Figure 9, it can be observed that the nine time series in the CBF dataset generally exhibit distinct segmented trend characteristics across the three classes. The experiment demonstrates the effectiveness of the method introduced in this paper for time series with various shapes and trends.

### 5.4. Experiment B on 4 UCR Datasets

Experiment B utilizes four datasets from the UCR Time Series Classification Archive. The fundamental information about these datasets is displayed in Table 4. After performing the CMAIG method on the experimental datasets, we calculate the CR using Equation (16), as shown in Table 5. It can be observed that the volume of the data from the four UCR datasets has significantly decreased following the application of CMAIG, with an average *CR* of 3.03%. This indicates that the volume of data after information granulation has been reduced to 3.03% of the original data scale.

In this experiment, we compare our method with those listed in reference [13] and use *F*-Measure as the evaluation metric for cluster validity. Table 6 presents the evaluation results of these clustering methods.

As shown in Table 6, on CBF dataset, the *F*-Measure of CMAIG_ECM_HC is higher than that of the other algorithms. On the Gun-Point dataset, the *F*-Measure of CMAIG_ECM_HC is slightly lower than the PWCA_HC algorithm but higher than the other six algorithms. On the Trace dataset, the *F*-Measure of CMAIG_ECM_HC is slightly lower than the DSA_DTW_HC algorithm but higher than the other six algorithms. On the synthetic_control dataset, the *F*-Measure of CMAIG_ECM_HC is lower than the 2D-NCR_HC algorithm but higher than the other six algorithms. We measure the performance of each algorithm across the four datasets using the average rank, with CMAIG_ECM_HC having an average rank of 1.75, which is the best among all the algorithms.

### 5.5. Experiment C on Real Stock Dataset

Experiment C utilizes a real stock dataset, comprising closing prices of 40 stocks from 3 January 2017, to 31 July 2020, spanning a total of 871 trading days. The stock price data are sourced from NetEase Finance [44]. The industries represented in this dataset primarily encompass eight sectors, including liquor (L), pharmaceuticals and biotechnology (PB), real estate (RE), banking (B), information technology (IT), fossil energy (FE), nonferrous metals (NM), and agriculture, forestry, animal husbandry, and fishing (AFHF).

**Figure 12:**
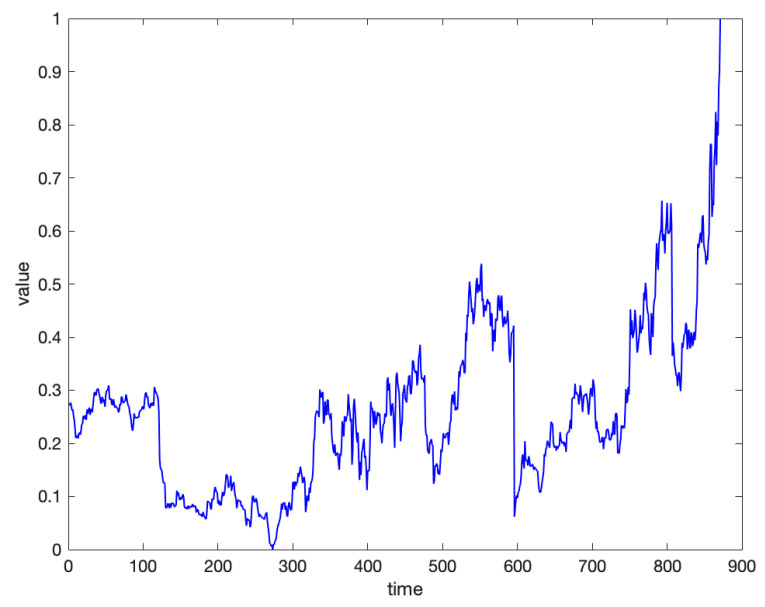
Example of a time series from the real stock dataset.

We perform normalization on the dataset, address the missing values, and select a time series from the dataset as an example. As shown in Figure 12, it can be observed that the trend of the daily closing price sequence over a longer time period exhibits distinct characteristics of high-frequency oscillations.

**Figure 13:**
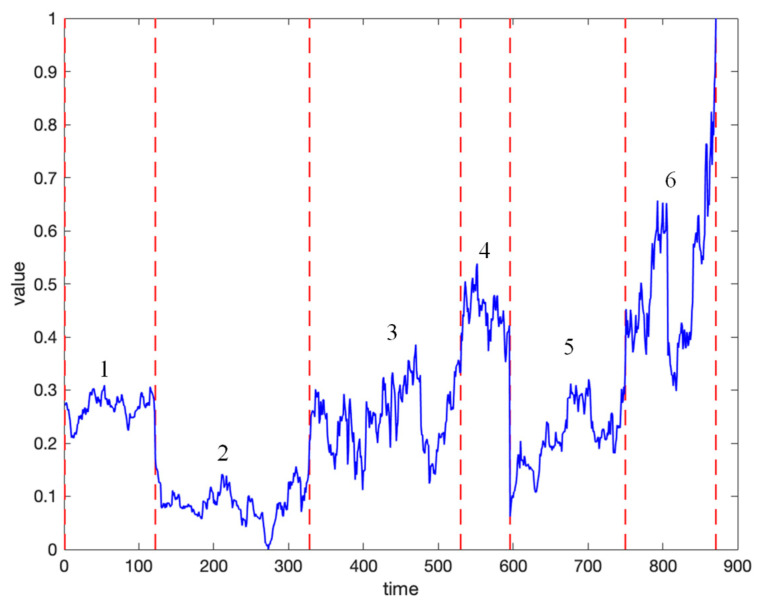
Example of a time series division using CMAIG.

Then, by applying CMAIG, the time series is divided into six information granules, each with significantly different structural features, as shown in Figure 13. This indicates that the proposed method can effectively divide and represent the oscillation characteristics of stock time series. After the information granulation processing, we obtain six CMIGs, resulting in a *CR* of 0.69%. This means that by applying CMAIG, efficient dimensionality reduction in time series can be achieved.

**Figure 14:**
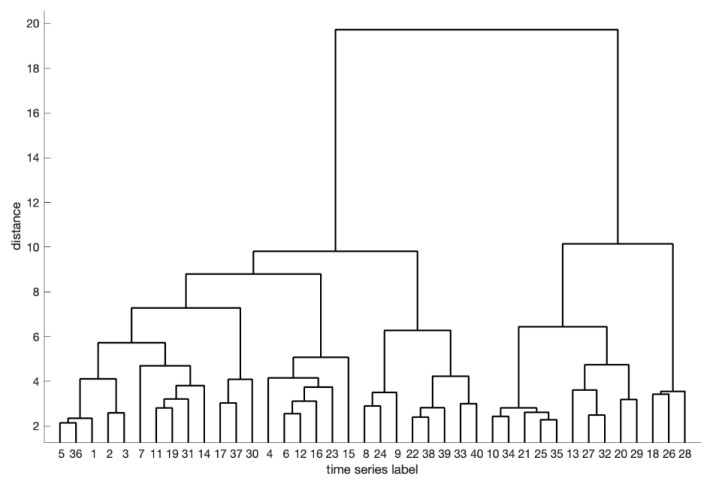
Clustering dendrogram of experiment C.

**Figure 15:**
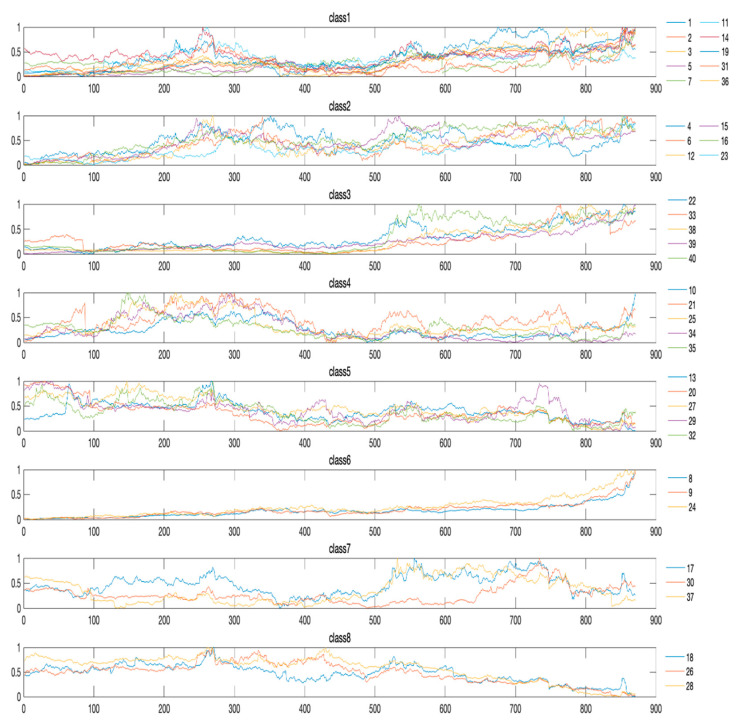
Eight clusters of 40 stock time series in experiment C.

After performing CMAIG on each stock time series in the experimental dataset, we apply the proposed similarity measurement method for hierarchical clustering, resulting in the dendrogram depicted in Figure 14. We set the clustering level threshold (the maximum distance between objects within a cluster) to six, which leads to the division of 40 stock time series into eight clusters. The specific class division results are presented in Figure 15 and Table 7.

From Figure 15, it can be observed that on the long-term Shanghai and Shenzhen A-share stock time series, the CMAIG_ECM_HC method can cluster the time series with a similar trend into the same cluster and assign those with different trends to different clusters. Overall, there are significant differences in the trend of these 40 stocks’ time series, and they exhibit clear phased trend characteristics. The following is a detailed description of each cluster.

Cluster 1 includes 10 time series, which generally show an upward trend with significant increases. During the first 400 trading days, there are considerable fluctuations without any clear gains or losses; from the 400th to the 500th trading day, the fluctuations are relatively small; after the 500th trading day, the fluctuations are larger and there is a marked upward trend.

Cluster 2 includes six time series, which overall exhibit an upward trend with substantial growth. During the first 200 trading days, they show a steady upward trend; from the 200th to the 500th trading day, there are significant fluctuations without any clear gains or losses; after the 500th trading day, they exhibit a trend of fluctuating increases.

Cluster 3 includes five time series, which overall show an upward trend with a significant increase. During the first 500 trading days, there are no noticeable fluctuations; after the 500th trading day, the fluctuations become larger, and a marked upward trend is observed.

Cluster 4 includes five time series, which overall exhibit an upward trend with a relatively small increase. During the first 300 trading days, there is a significant fluctuation with an upward trend; from the 300th to the 500th trading day, there is a marked downward trend; from the 500th to the 800th trading day, there are considerable fluctuations without any clear gains or losses; after the 800th trading day, there is a slight upward trend.

Cluster 5 includes five time series, which overall show a downward trend with a significant decrease. During the first 400 trading days, there is a marked downward trend; after the 400th trading day, the fluctuations are larger, but there are no clear gains or losses.

Cluster 6 includes three time series, which overall exhibit an upward trend with a substantial increase. During the first 800 trading days, they steadily rise but with a small increase; after the 800th trading day, they show a significant upward trend with no apparent fluctuations and a substantial increase.

Cluster 7 includes three time series, which overall exhibit a downward trend with a relatively small decrease. During the first 400 trading days, they experience significant fluctuations and a marked downward trend; from the 400th to the 500th trading day, the fluctuations are relatively small; after the 500th trading day, the fluctuations are larger, but there are no clear gains or losses.

Cluster 8 includes three time series, which overall show a downward trend with a significant decrease. During the first 500 trading days, there are considerable fluctuations with a downward trend and a relatively small decrease; after the 500th trading day, they steadily decline with a significant decrease.

Table 7 provides detailed information for the objects within each cluster, and it can be observed that the clustering results of these 40 stock’s time series do not have a clear correspondence with its industries. This means that the traditional method of stock selection based on industry types for investment portfolios is not reasonable.

Using the CMAIG_ECM_HC method proposed in this article, Shanghai and Shenzhen A-share stocks with similar stock price trend characteristics can be classified into the same cluster. This clustering result can provide a reference for investors when selecting stocks for investment in the A-share market. Investors can choose stocks from different clusters for portfolio investment according to their investment risk preferences to enhance returns and diversify risks.

## 6. Conclusions

In this article, we propose a new time series information granulation algorithm and its similarity measurement method. Firstly, an adaptive information granulation algorithm for time series based on the cloud model (CMAIG) is proposed. This method can adaptively divide a time series into several segments represented by normal clouds without pre-specifying the number of information granules. After using CMAIG, a granular sequence composed of cloud model information granules (CMIGs) is obtained. Then, a new similarity measurement method (CMAIG_ECM) is proposed for granular time series. A cloud model similarity measurement algorithm based on the expected curve (ECM) is used to calculate the similarity between any two CMIGs. The matching principles of CMIGs are defined, the concept and calculation method of time window coverage are introduced, and the similarity measurement method for cloud model sequences is proposed. Finally, some clustering experiments are conducted on the UCR datasets to verify the effectiveness of the proposed information granulation method and the similarity measurement method, and the algorithms are also practically applied to a real Shanghai and Shenzhen A-share stock dataset. In conclusion, the proposed methods can better represent the shapes and trends of time series, and achieve efficient dimensionality reduction, thereby leading to improved clustering results. In the future, we will explore and expand the applicable scope of the algorithm and develop different information granulation methods for time series with different characteristics. Moreover, we will consider improving the proposed method to detect and identify different tail regimes in time-series data.

## Figures and Tables

**Table 1:** Each information granule representation of a time series.

No.	(*t_s_*, *t_n_*, *Ex*, *En*, *He*)
1	(1, 27, −0.9092, 0.2627, 0.0916)
2	(27, 85, 0.9984, 0.3597, 0.2697)
3	(85, 128, −0.7827, 0.3814, 0.2201)

**Table 2:** CMIGs representation of two granular time series.

Granular Time Series *Q*		Granular Time Series *C*	
Segment	Representation of CMIG	Segment	Representation of CMIG
*q*_1_	(1, 27, −0.9092, 0.2627, 0.0916)	*c*_1_	(1, 17, −1.047, 0.4598, 0.3238)
*q*_2_	(27, 85, 0.9984, 0.3597, 0.2697)	*c*_2_	(17, 94, 0.7535, 0.3250, 0.0286)
*q*_3_	(85, 128, −0.7827, 0.3814, 0.2201)	*c*_3_	(94, 128, −1.1121, 0.4288, 0.3206)

**Table 3:** The matching result of CMIGs for two granular time series.

	Granular Time Series *Q^c^*		Granular Time Series *C^c^*	
No.	Segment	Representation of CMIG	Segment	Representation of CMIG
1	*q*_1_	(1, 27, −0.9092, 0.2627, 0.0916)	*c*_1_	(1, 17, −1.047, 0.4598, 0.3238)
2	*q*_1_	(1, 27, −0.9092, 0.2627, 0.0916)	*c*_2_	(17, 94, 0.7535, 0.3250, 0.0286)
3	*q*_2_	(27, 85, 0.9984, 0.3597, 0.2697)	*c*_2_	(17, 94, 0.7535, 0.3250, 0.0286)
4	*q*_3_	(85, 128, −0.7827, 0.3814, 0.2201)	*c*_2_	(17, 94, 0.7535, 0.3250, 0.0286)
5	*q*_3_	(85, 128, −0.7827, 0.3814, 0.2201)	*c*_3_	(94, 128, −1.1121, 0.4288, 0.3206)

**Table 4:** Description of four UCR datasets used in experiment B.

Dataset	Samples	Length	Classes
Gun-Point	50	150	2
Trace	100	275	4
synthetic_control	300	60	6
CBF	30	128	3

**Table 5:** *CR* after CMAIG on four UCR datasets.

Dataset	Length	Average Number of CMIGs	*CR* (%)
Gun-Point	150	3	2.00
Trace	275	3	1.09
synthetic_control	60	4	6.67
CBF	128	3	2.34

**Table 6:** *F*-Measure comparison of algorithms in experiment B.

Dataset	DTW_ HC	PLA_ DTW_HC	PAA_ DTW_HC	SAX_ DTW_HC	DSA_ DTW_HC	PWCA_ HC	2D-NCR_ HC	CMAIG_ ECM_HC
CBF	0.51(6)	0.51(6)	0.51(6)	0.56(5)	0.6(4)	0.72(3)	0.76(2)	0.84(1)
Gun-Point	0.61(4)	0.61(4)	0.61(4)	0.61(4)	0.61(4)	0.73(1)	0.69(3)	0.71(2)
Trace	0.48(8)	0.63(4)	0.61(5)	0.6(6)	0.82(1)	0.55(7)	0.76(3)	0.78(2)
synthetic _control	0.48(5)	0.4(7)	0.36(8)	0.48(5)	0.54(4)	0.68(3)	0.81(1)	0.72(2)
Avg Rank	5.75	5.25	5.75	5	3.25	3.5	2.25	1.75

DTW_HC: Hierarchical clustering algorithm based on dynamic time warping distance. PLA_DTW_HC: Hierarchical clustering based on piecewise linear approximation of dynamic time Warping distance. PAA_DTW_HC: Hierarchical clustering based on piecewise aggregate approximation of dynamic time warping distance. SAX_DTW_HC: Hierarchical clustering based on symbolic aggregate approximation of dynamic time warping distance. DSA_DTW_HC: Hierarchical clustering based on inverse splitting approximation of dynamic time warping distance. PWCA_HC: Hierarchical clustering based on cloud model segment approximation representation. 2D-NCR_HC: Hierarchical clustering based on two-dimensional normal cloud model representation. CMAIG_ECM_HC: Hierarchical clustering based on cloud model adaptive information granulation of cloud model expected curve similarity measurement (the algorithm proposed in this paper).

**Table 7:** Information of eight clusters of 40 stock time series in experiment C.

Class	Amount	Industry	Number of Companies
1	10	L	4
		PB	1
		RE	2
		B	1
		NM	1
		AFHF	1
2	6	L	1
		PB	1
		RE	2
		B	1
		IT	1
3	5	IT	1
		NM	1
		AFHF	2
4	5	PB	1
		IT	2
		NM	2
5	5	RE	1
		B	1
		FE	2
		NM	1
6	3	PB	2
		IT	1
7	3	B	1
		FE	1
8	3	AFHF	1
		B	1
		FE	2

## Data Availability

The data used to support the findings of this paper are available from the corresponding author upon reasonable request.
